# Novel Asleep Techniques for Intraoperative Assessment of Brain Connectivity

**DOI:** 10.3389/fneur.2021.687030

**Published:** 2021-06-28

**Authors:** Francesco Sala, Davide Giampiccolo, Luigi Cattaneo

**Affiliations:** ^1^Section of Neurosurgery, Department of Neuroscience, Biomedicine and Movement Sciences, University of Verona, Verona, Italy; ^2^CIMeC - Center for Mind/Brain Sciences, University of Trento, Trento, Italy

**Keywords:** intraoperative neurophysiological monitoring, motor evoked potentials, cortico-cortical evoked potentials, neuro-oncology – surgical, brain mapping

## Introduction

### State of the Art in Intraoperative Monitoring for Neurosurgery and Current Limitations

From the pioneering work of neurosurgeon-neuroscientists such as Otfried Foerster and Wilder Penfield, mapping of brain function using electrical stimulation has allowed to identify and spare motor behavior and language during awake surgery, producing the first cartographies of the brain cortex (1, 2). This has been refined in the early 2000s as neurosurgeons started routine subcortical awake mapping using white matter tracts as subcortical boundaries (3), with subsequent improvement in both functional (4) and surgical outcome (5, 6). The advantages of performing awake surgery when cognitive functions are at risk, should not be questioned, and we remark this should be performed whenever feasible. However, patients with pre-existing neurological deficits and/or inadequate neuropsychological profiles are not good candidates for awake surgery, and therefore must undergo asleep procedures. Notably, pediatric patients cannot undergo awake surgery, on one hand because of their scarce compliance (7), on the other hand because the immaturity of their motor system makes the cortex almost inexcitable using the traditional bipolar Penfield's 50/60 Hz technique (8, 9).

In patients who are poor candidates for awake surgery, developing methods to map cortico-cortical and cortico-subcortical connectivity under anesthesia is of primary importance since otherwise surgery will be performed blind to function, with higher risk of incurring into neurological deficits. Since the adoption of somatosensory evoked potentials (SEPs) in the ‘70s (10) and particularly with the development of the train-of-five motor evoked potential (MEP) technique (11), the field of intraoperative neurophysiological monitoring (IONM) has specifically addressed the issue of mapping and monitoring during asleep anesthesia. Standard protocols for motor mapping are nowadays available, offering reproducible, and reliable parameters to qualitatively and quantitatively predict outcome (12). This somehow differed from awake surgery, where neuropsychological tests and mapping protocols have a greater degree of variability. In brain tumor surgery IONM is particularly well-established for preservation of the corticospinal tract (13, 14), but asleep mapping and monitoring outside the corticospinal system is lacking. The aim of this opinion paper is to discuss two novel potential IONM techniques allowing to map and monitor functions beyond corticospinal motor function in the anesthetized setting, namely, (A) conditioning of motor output (15, 16) and (B) cortico-cortical evoked potentials (17, 18). These have been performed under total intravenous anesthesia using Propofol (100–150 μg/kg/min) and Fentanyl (1 μg/kg/min) in continuous infusion, and avoiding muscle relaxants after intubation.

### Potential Novel Intraoperative Measures of Brain Connectivity in the Anesthetized Patient

#### Conditioning of Corticospinal Output

**Figure 1:**
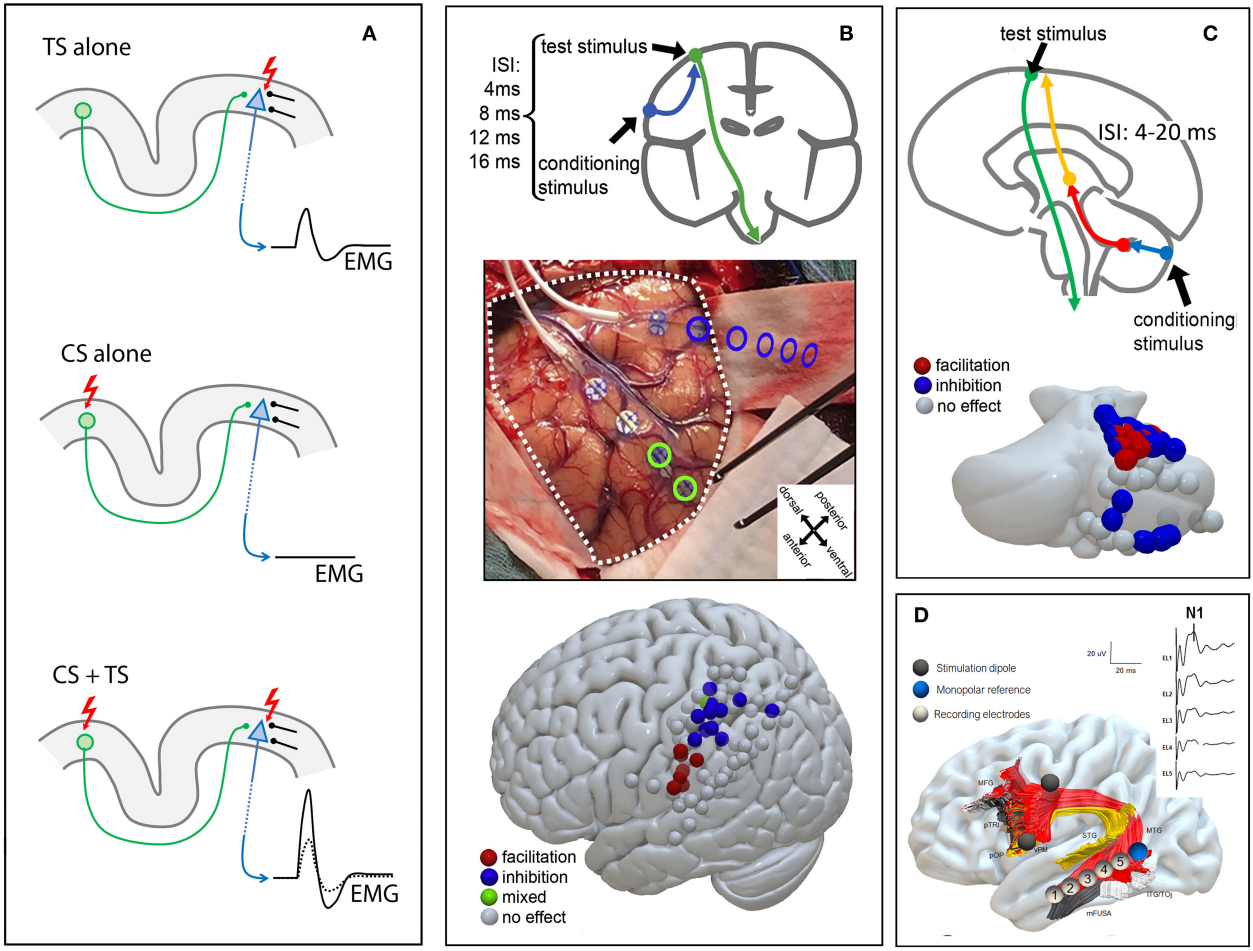
Schematic CS/TS and ccEP paradigms. **(A)** *Upper panel* TS stimulation alone induces an MEP; *middle panel* CS stimulation alone does not evoke an MEP; *lower panel* combined CS + TS stimulation leads to increased MEP amplitude. *CS, conditioning stimulus; TS, test stimulus* **(B)** Parieto-motor stimulation protocol: a CS stimulus is applied over the posterior parietal cortex from 4 to 16 ms before a TS on M1. A dorso-ventral posterior parietal distinction between MEP inhibition (dorsal) and MEP facilitation (ventral) is shown. The picture is modified from (15) with permission *CS, conditioning stimulus; TS, test stimulus* **(C)** Cerebello-motor stimulation protocol: direct cerebellar precedes transcranial electrical stimulation with an inter-stimulus interval (ISI) of 4–20 ms, causing MEP inhibition (blue) or facilitation (red). **(D)** ccEPs for the arcuate fasciculus: strip electrodes are placed intraoperatively at cortical termination of the arcuate fasciculus after preoperative testing. ccEPs are recorded from the middle temporal cortical termination of the arcuate fasciculus (shown on the right side). Segments of the arcuate fasciculus are dissected according to cortical termination (19). *MFG, middle frontal gyrus; pTRI, pars triangularis; pOP, pars opercularis; vPM, ventral premotor cortex; STG, superior temporal gyrus; MTG, middle temporal gyrus; ITG, inferior temporal gyrus; TOj, temporo-occipital junction; mFUSA, middle fusiform gyrus*.

The conditioning stimulus (CS)/test stimulus (TS) paradigms have been used widely in experimental and clinical neurophysiology to investigate functional connectivity between two regions of the nervous system. In CS/TS paradigms (illustrated in panel A of Figure 1), a suprathreshold stimulus (TS) is delivered to the motor cortex, thus evoking a motor potential (MEP) of a given amplitude. The CS, of intensity comparable to that of the TS, is delivered to a region that is supposedly connected to the motor cortex but is not itself a source of corticospinal output. Therefore, the CS alone does not produce a MEP. However, when the CS precedes the TS, the MEP obtained by the conditioned TS may be different (i.e., increased or decreased MEP amplitude) from that obtained by TS alone. Whenever such remote effects of CS on TS occur, they are taken as evidence of functional connectivity between the site of application of the CS and that of the TS. The CS-TS interactions are generally specific for given inter-stimulus intervals (ISI), i.e., the time interval between CS and TS. Conditioning effects of the CS over the TS at short ISIs is generally thought to be informative of the underlying anatomical connections: interactions at short ISIs indicate direct cortico-cortical connections.

CS/TS paradigms are commonly explored using non-invasive brain stimulation, namely transcranial magnetic stimulation (TMS) applied with two different coils over the scalp, one delivering the CS and the other delivering the TS over the motor cortex (20). These paradigms have been extensively reviewed by Koch (21). The modulatory effect of CS can be excitatory or inhibitory, therefore increasing or decreasing MEP amplitude compared to TS alone. However, as MEP amplitude can physiologically vary between two identical stimuli, it is important to repeat both TS stimulations and CS+TS stimulation. Therefore, the comparison between CS+TS MEPs and MEPs to TS alone cannot be done between single MEPs, but must be performed between groups of conditioned (CS+TS) MEPs and of baseline (TS alone) MEPs.

The descending corticospinal volley evoked by stimulation of the motor cortex has different components, separated in time. The earliest volley is referred to as direct, or “D” wave and is due to direct activation of corticospinal axons. The later components, known as indirect or “I” waves originate from stimulation of neurons that in turn project onto the corticospinal neurons, which are therefore activated trans-synaptically (22). It is important to note that for the TS to be susceptible to modulation by the CS, it must produce a corticospinal volley containing I-waves, as the D-wave cannot be modulated by any afferents to the corticospinal neuron because it is generated downstream of any point of integration of inputs from cortico-cortical afferents. Indeed, direct cortical stimulation and intraoperative transcranial electrical stimulation are known to produce I-waves (23–25).

We propose that the CS/TS approach can be successfully performed also by means of direct cortical stimulation in the intraoperative setting, and therefore potentially be used for assessing and potentially monitoring the integrity of specific brain and spine connections, as demonstrated by two recent works from our group, on parietal-motor connectivity (15) and on cerebello-motor connectivity (16).

In the first **work** we described the successful use of a CS/TS paradigm in the intraoperative setting to explore putative direct parietal-motor connectivity (15) (Figure 1B). Briefly, two strip electrodes were deployed, one over the parietal cortex (CS) and one over the motor cortex (TS). Conditioning stimuli in the parietal cortex were always delivered in a short train of 2 stimuli at 250 Hz and of 0.5 ms duration while test stimulus varied from a single stimulus to a train of 3 stimuli at 250 Hz and 0.5 ms duration according to the individual patient's cortical excitability. Stimulation intensity for conditioning and test stimulation was always the same (15–35 mA). Prior to all experimental stimulations we acquired blocks of conditioning stimuli alone, verifying that no MEP could be observed from test stimulation.

Such functional connectivity has been abundantly described by means of dual coil TMS in healthy participants (20, 26). In our study we highlighted the presence of conditioning effects from CS applied *directly* to the posterior parietal cortex on the TS applied *directly* to the ipsilateral upper limb motor cortex, in 17 anesthetized oncological patients during surgical resection. The conditioning effects on the TS were specific for both timing and anatomical localization of the CS. The effects appeared at short ISIs (4–20 ms) with the earliest effective ISI depending on the anatomical proximity of the parietal stimulation electrode to the motor cortex: short ISIs were efficient when the CS was delivered near to the motor cortex. The use of trains of stimuli to stimulate the motor cortex renders timing of the CS/TS ISI difficult. In general we considered, for the sake of timing, the last stimulus of the train as the one that is effective in triggering the efferent corticofugal volley (27). Anatomical specificity was clearly evident in the spatial clustering of CS sites with excitatory effects in the inferior parietal lobule and of CS sites with inhibitory effects in the superior parietal lobule. Note that focality of CS stimulation was granted by the use of bipolar stimulation between adjacent electrodes of a stimulating strip.

In the second **work** we tested the effects of CS applied to the cerebellar cortex onto corticospinal excitability (16), tested by TS applied *transcranially* to target the upper limb motor cortex (Figure 1C) in 10 anesthetized patients undergoing posterior fossa tumor surgery. Our experimental paradigm is inspired by a well-established CS+TS technique referred to as “cerebellar inhibition” in which the cerebellum and the motor cortex are stimulated by TMS (28, 29). Briefly, conditioning stimuli on the cerebellar cortex were delivered in a short train of 2–5 stimuli at 250 Hz and of 0.5 ms duration with an intensity of 15–25 ms. Test stimuli varied from a train of 2–5 stimuli at 250 Hz and 0.5 ms duration with stimulation intensity of 15–35 mA. Our results showed that CS cerebellar stimuli conditioned at short ISIs the corticospinal excitability, with a significant anatomical specificity: cerebellar CS exerted conditioning effects on the hand corticospinal system when applied to regions of the cerebellar cortex in the anterior and posterior lobule that are known to contain hand representations (30, 31).

#### Cortico-Cortical Evoked Potentials in the Anaesthetized Patient

While the rationale for describing brain connectivity by means of ccEPs was first discussed by Lord Adrian (32), the clinical use of cortico-cortical evoked potentials was first pioneered by Matsumoto (33). In ccEPs, one of two cortical terminations of a white matter tract is stimulated electrically, and cortical evoked activity is recorded at the other termination in the form of evoked potentials. Twenty to 120 raw traces are conventionally averaged, similarly to cortically recorded somatosensory evoked potentials. ccEPs show two components, an N1 between 20 and 30 ms (33, 34) and a second, later component peaking at 100–150 ms (33), though some authors claim this later component could represent epileptogenic activity instead (35). CcEPs latency should reflect fiber myelination and length (33, 36). Moreover, there is strong evidence for directionality in the evoked potentials (37, 38). The recording of ccEPs is potentially applicable to the whole cerebral cortex and transcends language function (39, 40). However, clinical use of this technique has been historically mainly related to language function.

The recording of ccEPs is generally limited to awake patients, because of (a) the suppression of neural activity due to anesthesia (41), and (b) the chance to identify location for strip electrodes placement using functional mapping (33, 42). However, their use is of potential interest in monitoring white matter integrity also in the asleep patient. In a recent work in a cohort of 9 patients with tumors in the left perisylvian area who could not undergo awake surgery, we recorded ccEPs of the arcuate fascicle in anesthetized patients undergoing tumor surgery (18). Results indicated that (a) reliable potentials of small amplitude can be obtained from the arcuate fasciculus also under anesthesia and that (b) strip electrode placement can be made more effective by combining tractographic MR information and presurgical neuronavigated TMS (nTMS). Results in the asleep setting resembled those in the awake setting: an N1 potential with a latency of 21 ms was shown, together with an earlier positive potential peaking at 12 ms. In our series, evoked potentials clustered in the middle temporal gyrus while stimulation mainly covered the ventral premotor cortex. Although the arcuate fasciculus has cortical terminations over superior, middle and inferior temporal gyri (43, 44), such selectivity may be justified by a layered distribution of its components, particularly in a ventral/dorsal fashion (19, 44). Indeed, ccEP responses for this tract may support this, since location for optimal recorded responses varies according to the stimulated gyrus in the frontal lobe (45). Moreover, the inferior temporal gyrus components of the arcuate fasciculus have not been extensively investigated in this study, which is another limitation to be taken into account.

## Conclusion and Limitations

Asleep surgery without mapping is blind to function and therefore at highest risk of inducing neurological deficits. We believe it is an ethical responsibility to raise awareness of this issue. Therefore, the work presented here lies on this foundation and the attempt to predict, and therefore prevent, neurological deficits in patients who are not good candidates to an awake craniotomy.

However, to be useful in clinical practice these techniques require to (a) be standardized; (b) be deterministic, i.e., allow predictions in individual patients; (c) have a strong predictive value of a given clinical/behavioral aspect. The phenomena that we have described do not satisfy any of the above criteria, therefore further research is needed before a clinical use, if any, can be proposed. Points (a) and (b) require extensive tests for standardization and reproducibility. Regarding point (c), we believe that cortico-motor CS/TS paradigms should be tested also from a constellation of other areas that project the motor cortex, namely the premotor, supplementary motor and somatosensory cortices and their perioperative changes need to be correlated with behavior such as skilled movement and sensorimotor behavior in general. The cerebello-motor CS/TS paradigm's predictive value should be tested specifically on the pediatric population undergoing posterior fossa surgery. This is of overriding importance, as individual age-associated myelination and axonal length (9) may imply significant changes in the optimal parameters (ISI, stimulation intensity) for cerebello-cortical modulation. Similarly, ccEPs for language connectivity should be tested for their predictive value in perioperative language disorders.

## Author Contributions

FS, DG, and LC: conceptualization and writing - original draft. FS: funding acquisition. FS and LC: supervision. All authors contributed to the article and approved the submitted version.

## Conflict of Interest

The authors declare that the research was conducted in the absence of any commercial or financial relationships that could be construed as a potential conflict of interest.

## Abbreviations

AF: arcuate fasciculus
CCEPs: Cortico-cortical evoked potentials
CS: conditioning stimulus
CST: cortico-spinal tract
DES: direct electrical stimulation
DTI: diffusion tensor imaging
DWI: diffusion weighted imaging
ECoG: electrocorticography
EEG: electroencephalography
EMG: electromyography
HARDI: high angular resolution diffusion imaging
ISI: interstimulus interval
IONM: intraoperative neurophysiological monitoring
MEPs: motor evoked potentials
MFG: middle frontal gyrus
mFUSA: middle fusiform gyrus
MTG: middle temporal gyrus
pOP: pars opercularis
pTRI: pars triangularis
STG: superior temporal gyrus
TOj: temporo-occipital junction
To5: train-of-five stimulation
TS: test stimulus
vPM: ventral premotor cortex
ITG: inferior temporal gyrus.
